# Supplementary material for: Titin-Related Dilated Cardiomyopathy: The Clinical Trajectory and the Role of Circulating Biomarkers in the Clinical Assessment
Source: Diagnostics (Basel). 2021 Dec 22;12(1):13. doi: 10.3390/diagnostics12010013 (PMC8775078; doi:10.3390/diagnostics12010013)
Supplement: Supplementary file 1 [file diagnostics-12-00013-s001.zip › diagnostics-1498026-supplementary.pdf]

Supplementary Table S1. Probands with titin truncating variants identified in the study cohort

Proband	**Metatranscript NM_001267550.2**	**Protein NP_001254479.2**	***TTN* variant type**	**Exon meta-transcript**	**Band**	**PSI**	**No. of relatives with carrier status**
1	c.414C>A [n]	p.Tyr138Ter	nonsense	4	Z-disc	1.00	2
2	c.1478C>A #	p.Ser493Ter	nonsense	9	Z-disc	1.00	8
3	c.12704C>A	p.Ser4235Ter	nonsense	48	I-band	1.00	0
4	c.13058delC	p.Pro4353GlnfsTer14	frameshift	48	I-band	1.00	2
5	c.41923dup [n]	p.Thr13975AsnfsTer4	frameshift	228	I-band	1.00	1
6	c.43360G>T	p.Glu14454Ter	nonsense	235	I-band	1.00	2
7	c.43582A>T #	p.Lys14528Ter	nonsense	236	I-band	1.00	1
8	c.44146dup [n]	p.Gln14716ProfsTer4	frameshift	238	I-band	1.00	0
9	c.44249del [n]	p.Asn14750MetfsTer14	frameshift	239	I-band	1.00	0
10	c.44281+1G>A	n/a	splice site	239 near	I-band	1.00	1
11	c.44410G>T [n]	p.Glu14804Ter	nonsense	240	I-band	1.00	0
12	c.47192del [n]	p.Arg15731LeufsTer2	frameshift	252	A-band	1.00	0
13	c.50083C>T	p.Arg16695Ter	nonsense	240	I-band	1.00	1
14	c.53206C>T #	p.Arg17736Ter	nonsense	277	A-band	1.00	1
15	c.53259del	p.Lys17753AsnfsTer7	frameshift	277	A-band	1.00	0
16	c.54652C>T [n]	p.Arg18218Ter	nonsense	282	A-band	1.00	1
17	c.54652C>T [n]	p.Arg18218Ter	nonsense	282	A-band	1.00	1
18	c.56751_56752del #	p.Gly18918ValfsTer17	frameshift	291	A-band	1.00	2
19	c.57769C>T	p.Arg19257Ter	nonsense	295	A-band	1.00	2
20	c.61197_61198insAT [n]	p.Gly20400MetfsTer6	frameshift	304	A-band	1.00	2
21	c.62337_62340del [n]	p.Thr20780SerfsTer32	frameshift	304	A-band	1.00	0
22	c.63025C>T #	p.Arg21009Ter	nonsense	304	A-band	1.00	4
23	c.64473T>A	p.Tyr21491Ter	nonsense	309	A-band	1.00	0
24	c.68449C>T #	p.Arg22817Ter	nonsense	322	A-band	1.00	2
25	c.70496dup	p.Leu23499PhefsTer3	frameshift	326	A-band	1.00	1
26	c.70540G>T #	p.Glu23514Ter	nonsense	326	A-band	1.00	0

27	c.73508del	p.Asn24503IlefsTer24	frameshift	326	A-band	1.00	0
28	c.73734_73735insCCAC [n]	p.Lys24579ProfsTer11	frameshift	326	A-band	1.00	1
29	c.78439C>T #	p.Gln26147Ter	nonsense	326	A-band	1.00	0
30	c.78979C>T	p.Arg26327Ter	nonsense	326	A-band	1.00	2
31	c.80487delT #	p.Ile26829MetfsTer15	frameshift	326	A-band	1.00	2
32	c.81010C>T #	p.Gln27004Ter	nonsense	326	A-band	1.00	5
33	c.81391A>T #	p.Lys27131Ter	nonsense	326	A-band	1.00	0
34	c.84504dup	p.Ser28169IlefsTer12	frameshift	326	A-band	1.00	2
35	c.85408_85409delCT [n]	p.Leu28470PhefsTer22	frameshift	326	A-band	1.00	2
36	c.86076dup #	p.Ser28693IlefsTer2	frameshift	326	A-band	1.00	1
37	c.87355delG #	p.Ala29119LeufsTer17	frameshift	328	A-band	1.00	3
38	c.87355delG #	p.Ala29119LeufsTer17	frameshift	328	A-band	1.00	1
39	c.87355delG	p.Ala29119LeufsTer17	frameshift	328	A-band	1.00	0
40	c.87355delG	p.Ala29119LeufsTer17	frameshift	328	A-band	1.00	0
41	c.87757del #	p.Ser29253AlafsTer18	frameshift	329	A-band	1.00	1
42	c.88703_88704del	p.His29568LeufsTer7	frameshift	332	A-band	1.00	4
43	c.92199dup #	p.Asn30734GlnfsTer17	frameshift	339	A-band	1.00	3
44	c.93166C>T #	p.Arg31056Ter	nonsense	340	A-band	1.00	3
45	c.93166C>T	p.Arg31056Ter	nonsense	340	A-band	1.00	2
46	c.101227C>T	p.Arg33743Ter	nonsense	358	M-band	1.00	2

Legend: PSI , proportion spliced-in index is the proportion of transcripts that include a given exon of *TTN,* reflecting the exon usage in human LV [6]. Orange colour denotes probands who were excluded from the study due to concomitant likely pathogenic variants in other DCM-related genes (see Supplementary Table S2); #, probands described by us in [3]; [n], novel variants.

Supplementary Table S2. Likely pathogenic variants identified in other DCM-related genes in the cohort of titin truncating variant carriers.

Proband	Gene	Concomitant Variant	Titin truncating variant
23	*SCN5A*	NM_198056.3:c.5527G>T(p.Val1843Leu)	NM_001267550.2: c.64473T>A(p.Tyr21491Ter)
29	*MYH7*	NM_000257.3: c.709C>T(p.Arg237Trp)	NM_001267550.2: c.78439C>T (p.Gln26147Ter)
35	*TNNT2*	NM_001001430.2: c.133G>A(p.Glu45Lys)	NM_001267550.2: c.85408_85409delCT (p.Leu28470PhefsTer22)

Supplementary Table S3. Additional Baseline Clinical Characteristics of *TTN* Variant Carriers at Initial Visit.

	All N= 108	DCM N= 70 (64.8%)	non-DCM N= 38 (35.2%)	p
Risk factors				
Alcohol abuse, n=101	6 (5.9%)	6/65 (9.2%)	0	0.086
Chemotherapy	0	0	0	NA
Obesity	28 (25.9%)	23 (32.9%)	5 (13.2%)	0.026
Comorbidities				
Coronary artery disease	5 (4.6%)	4 (5.7%)	1 (2.6%)	**0.655**
Hypertension	27 (25.0%)	21 (30.0%)	6 (15.8%)	0.103
Diabetes mellitus	4 (3.7%)	3 (4.3%)	1 (2.6%)	1.00
Medication (n=105)				
β-blocker	60 (57.1%)	55 (82.1%)	5 (13.2%)	**<0.001**
ACE-I or ARB	64 (60.9%)	59 (88.1%)	5 (13.2%)	<0.001
MRA	39 (37.1%)	39 (58.2%)	0	**<0.001**

Legend: Number of subjects is expressed as n (%). Alcohol abuse was defined as a self-reported history of daily alcohol intake of more than 2U/day over a period longer than 3 years. Obesity was defined as body mass index >30 kg/m^2. ACE-I, angiotensin converting enzyme inhibitor; ARB, angiotensin receptor blocker; MRA, mineralocorticoid receptor blockers.

Supplementary Table S4. Penetrance of disease indicators estimated by Kaplan-Meier method in all *TTN* truncating variant carriers (n=108).

Disease indicator	Age				
	20y	30y	40y	50y	60y
LVD	8.4%	25.9%	47.4%	68.0%	78.1%
severe LVSD	3.7%	13.3%	31.2%	49.6%	63.1%
HF	3.7%	12.2%	30.4%	53.6%	67.0%
NT-proBNP	2.7%	11.7%	26.5%	58.1%	78.5%
VA	0%	10.2%	26.6%	49.8%	67.4%
AA	0.9%	5.5%	14.8%	25.5%	55.8%
AVB	0%	3.5%	6.1%	13.6%	36.3%
MVA	0%	1.2%	4.0%	11.1%	28.7%
esHF	1.9%	3.9%	6.3%	9.7%	22.4%
LBBB	0%	0%	2.7%	8.6%	25.3%
hs-cTnT	0%	1.4%	3.2%	5.3%	14.9%

Legend: AA, atrial arrhythmia; AVB, atrioventricular block; esHF, end-stage heart failure; HF, heart failure; hs-cTnT, high-sensitivity cardiac troponin T concentration >14 ng/l; LBBB, left bundle branch block; LVD, left ventricular dysfunction; LVSD, left ventricular systolic dysfunction; MVA, malignant ventricular arrhythmia; NT-proBNP, N-terminal pro-B-type natriuretic peptide serum concentration >125 pg/ml; VA, ventricular arrhythmia.

Supplementary Table S5. Baseline clinical characteristics of the study cohort of *TTN* truncating variant carriers and the cohort of DCM-related *LMNA* variant carriers, described previously [17].

	*TTN*tv carriers N= 108	*LMNA* carriers N=53	P
Age, years	39.7±15.5	33.2±12.4	0.009
Men	67 (62.0%)	31 (58.5%)	0.665
Probands	43 (39.8%)	21 (39.6%)	0.981
Symptoms			
Heart failure	54 (50.0%)	19 (35.9%)	0.090
NYHA class ≥3	14 (13.0%)	7 (13.2%)	0.965
Arrhythmias and CCD			
Atrial arrhythmias	25 (23.1%)	21 (42%)	0.015
nsVT	42 (39.6%)	30 (60%)	0.017
LBBB	11 (10.2%)	8 (17.0%)	0.233
AV block (≥1st degree)	15 (13.9%)	31 (59.6%)	<0.001
Echocardiography			
LVEF <50%	66 (61.1%)	19 (35.8%)	0.003
LVEF, %	43.5 ± 13.8	50.5±16.2	0.005
LVEDD, mm	58.3 ± 9.6	53.7±8.7	0.004
Biomarkers in stable phase			
hs-cTnT, ng/l	4.4 [<3.0; 8.3]	13.6 [7.0; 23.9]	<0.001
hs-cTnT >14 ng/l	9 (10.0%)	20 (47.6%)	<0.001
NT-proBNP, pg/ml	244 [76; 1225]	161 [73; 684]	0.263
NT-proBNP >125 pg/ml	47 (65.3%)	23 (54.8%)	0.266
Diagnoses			
DCM	70 (64.8%)	22 (41.5%)	0.005
indeterminate CM	13 (12.0%)	21 (39.6%)	<0.001
no CM	25 (23.2%)	10 (18.9%)	0.536
Implantable devices			
PM for bradyarrhythmias	5 (4.6%)	19 (35.8%)	<0.001
CRT-D	2 (1.8%)	3 (5.7%)	0.332
ICD/CRT-D	14 (13.0%)	16 (30.2%)	0.008

Legend: Number of subjects is expressed as n (%). Continuous variables are shown as mean ± standard deviation or median and quartiles [Q1:25th; Q2:75th percentiles]. AV block, atrioventricular block; CCD, cardiac conduction defect; CM, cardiomyopathy; CRT-D, cardiac resynchronization therapy defibrillator; DCM, dilated cardiomyopathy; hs-cTnT, high-sensitivity cardiac troponin T serum concentration; ICD, implantable cardioverter defibrillator; LBBB, left bundle branch block; *LMNA*, lamin A/C gene; LVEDD, left ventricular end-diastolic

dimension; LVEF, left ventricular ejection fraction; nsVT, non-sustained ventricular tachycardia; NT-proBNP, N-terminal pro-B-type natriuretic peptide serum concentration; NYHA class, New York Heart Association functional class; PM, pacemaker; *TTN*tv, titin truncating variant.

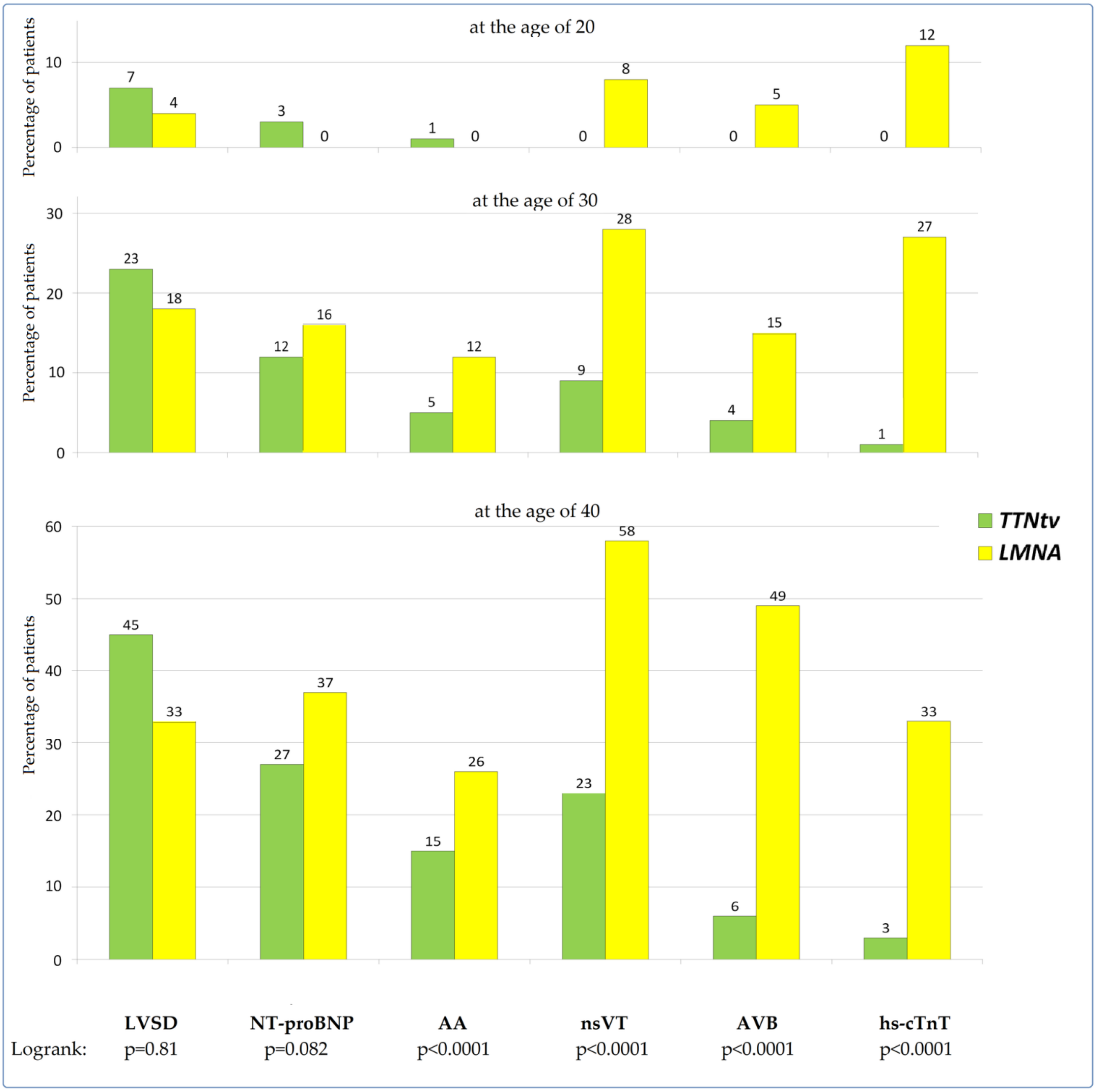

Supplementary Figure S1. Comparison of penetrance of disease indicators at the age of 20, 30, and 40 years, respectively, estimated by Kaplan-Meier method between the study cohort of *TTN* truncating variant carriers and the cohort of DCM-related *LMNA* variant carriers, described previously [17]. Legend: AA, atrial arrhythmia; AVB, atrioventricular block; hs-cTnT, high-sensitivity cardiac troponin T concentration >14 ng/l; *LMNA*, lamin A/C gene; LVSD, left ventricular systolic dysfunction; nsVT, non-sustained ventricular tachycardia; NT-proBNP, N-terminal pro-B-type natriuretic peptide serum concentration >125 pg/ml; *TTN*tv, titin truncating variants.
